# Safety of totally implantable venous access devices and peripherally inserted central catheters in hematological malignancies patients: a meta-analysis

**DOI:** 10.3389/fonc.2025.1679363

**Published:** 2025-10-31

**Authors:** Meier Gu, Xiaguang Huang

**Affiliations:** Department of Hematology and Oncology, Ningbo University Affiliated Yangming Hospital, Yuyao, China

**Keywords:** peripherally inserted central catheters, totally implantable venous access ports, hematologic malignancies, infection, meta-analysis

## Abstract

**Background:**

The use of totally implantable venous access devices (TIVADs) and peripherally inserted central catheters (PICCs) are the two options for patients receiving chemotherapy for hematologic malignancies. However, it remains unclear which approach yields superior patient outcomes. This meta-analysis aimed to compare the efficacy of TIVAPs and PICCs in patients undergoing chemotherapy for hematologic malignancies.

**Methods:**

A comprehensive literature search was conducted in PubMed, Embase, Cochrane Library, Wanfang, and China National Knowledge Infrastructure (CNKI) to identify available articles comparing the effect of TIVADs and PICCs. Statistical analyses were performed using RevMan 5.3 and STATA 12.0, with odds ratios (OR) and 95% confidence intervals (CI) used as effect indicators.

**Results:**

A total of 10 studies, including 784 patients (386 in the TIVAD group and 398 in the PICC group), met the eligibility criteria. The meta-analysis results demonstrated that compared with PICCs, TIVAPs were associated with lower significantly risks of infection (OR: 0.21, 95% CI: 0.11-0.40), catheter occlusion (OR: 0.31, 95% CI: 0.13-0.77), phlebitis (OR: 0.16, 95% CI: 0.06-0.42), and catheter dislodgement (OR: 0.25, 95% CI: 0.08-0.76) compared to PICCs. However, there was no significant difference between the two devices in terms of thrombosis risk (OR: 0.37, 95% CI: 0.10-1.41).

**Conclusion:**

This meta-analysis suggests a potential association between TIVAPs and a lower risk of complications compared with PICCs in patients with hematologic malignancies undergoing chemotherapy.

## Introduction

Patients with hematologic malignancies, such as leukemia, lymphoma, and multiple myeloma, often require prolonged and recurrent intravenous chemotherapy, anti-infective treatment, and blood product transfusions (1, 2). Since these treatment procedures involve the infusion of chemotherapy drugs, peripheral venipuncture can not only cause phlebitis but also vein damage, thereby making long-term treatment more difficult (3). To ensure safe and effective drug administration while minimizing the discomfort of repeated venipunctures, a central venous access device (CVAD) is widely utilized in clinical practice (4).

Currently, two primary types of CVADs are commonly employed for chemotherapy in patients with hematologic malignancies: peripherally inserted central catheters (PICCs) and totally implantable venous access devices (TIVADs) (5, 6). PICCs are inserted through peripheral veins, such as the basilic, cephalic, or median cubital veins, and advanced into the central circulation (7). This method is relatively simple and does not require surgical intervention; furthermore, the device can be used immediately after insertion. However, the external catheter component increases the risk of infection and thrombosis. Additionally, patients with PICCs require regular maintenance, including flushing and dressing changes, to minimize complications (8, 9). In contrast, TIVADs are fully implanted venous access systems, with the port placed subcutaneously and the catheter positioned in the superior vena cava (10). Because TIVADs do not have an external component when not in use, they carry a lower risk of infection and require minimal maintenance. Furthermore, TIVADs can remain in place for extended periods, thus reducing the inconvenience of frequent catheter replacement during long-term treatment. The subcutaneous placement of the port also allows patients greater mobility and improved quality of life (11). However, the implantation procedure requires surgery, involves higher initial costs, and may be associated with complications such as subcutaneous infections, thrombosis, or catheter-related issues (12).

Although both PICCs and TIVADs are widely used in clinical practice, their respective advantages and limitations remain debated (13). Some studies suggest that TIVADs are superior in reducing infection and thrombosis rates, prolonging catheter retention, and enhancing patient satisfaction (14). Conversely, other studies argue that PICCs offer greater flexibility and eliminate surgical risks. Therefore, this study aimed to conduct a meta-analysis to compare the efficacy and safety of TIVADs versus PICCs in patients receiving chemotherapy for hematologic malignancies, thus providing evidence-based guidance for clinical decisions.

## Methods

The meta-analysis is conducted in accordance with the Preferred Reporting Items for Systematic Evaluation and Meta‐Analysis (PRISMA) (15).

### Search strategies

A comprehensive literature search was conducted across multiple databases, including PubMed, Embase, Cochrane Library, Wanfang, and China National Knowledge Infrastructure (CNKI), to identify studies published up to February 2025 on the application of peripherally inserted central catheters (PICC) and totally implantable venous access devices (TIVAD) in patients undergoing chemotherapy for hematologic malignancies. The search strategy was consisted of free text terms and Medical Subject Headings, such as “PICC”, “peripherally inserted central catheter”, “totally implantable vascular access device”, “PORT”, “TIVAD”, “hematologic malignancies”, and “chemotherapy”. No language limitation was set during the literature search. An additional relevant search was performed by manually searching the references of eligible studies or reviews.

### Eligibility criteria

The following inclusion criteria were adopted: (1) Population: patients receiving chemotherapy for hematologic malignancies; (2) Intervention and comparison: comparing the effect of TIVAD and PICC; (3) Outcome: infection, catheter occlusion, phlebitis, catheter dislodgement, and the thrombosis; (4) Study design: randomized controlled trials (RCTs) or case-controls studies. Articles with the following exclusion criteria were eliminated: (1) duplicate publications; (2) meta-analyses, conference articles, case reports, and reviews; (3) articles without available data.

### Data extraction and quality assessment

Data collection and extraction were conducted using a predesigned form. Disagreements were resolved through discussion. The following data were extracted for each included study: first author’s name, publication date, patient age, gender, sample size, and outcomes. The Cochrane risk of bias toll was used to evaluate the methodological quality and risk of bias of included RCTs. The process was conducted by two researchers separately, and differences were resolved through discussion.

### Statistical analysis

Data analysis was performed using RevMan 5.3 and STATA 12.0. The dichotomous variables used odds ratio (OR) as the effect indicator. All effect sizes were presented with 95% confidence intervals (CI). The I^2^ statistic and Cochran’s Q test were used to assess heterogeneity among studies. A I^2^ statistic values of < 25, 25-75%, and >75% indicate low, moderate, and high levels of heterogeneity, respectively. Substantial heterogeneity (I^2^>50%) was identified, a random-effects model was used to analysis, and we also conducted a sensitivity analysis to examine the source of heterogeneity. A P value <0.05 is taken to indicate statistical significance.

## Results

### Search results and study characteristics

**Figure 1:**
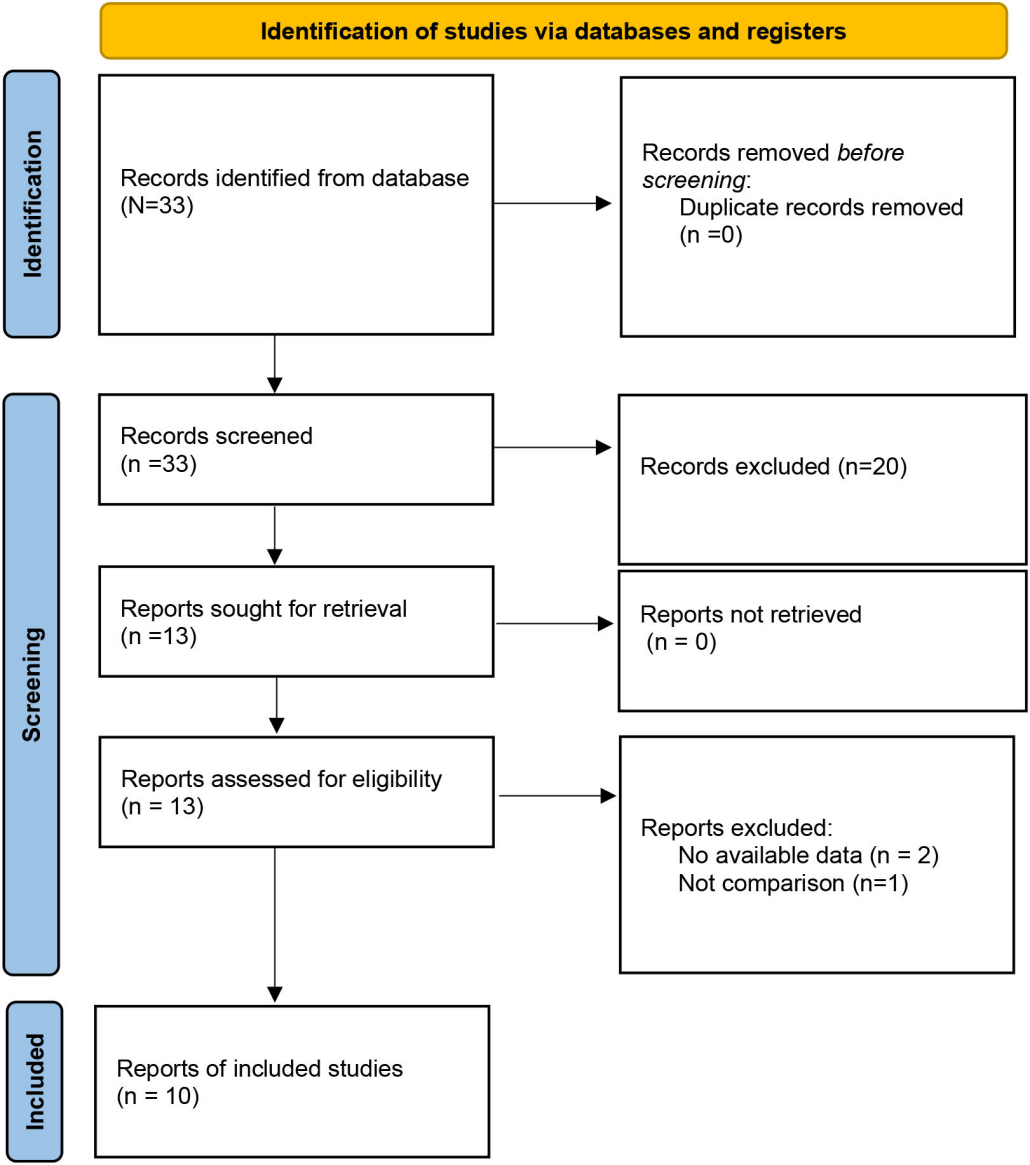
Study flowchart.

A total of 33 studies were initially retrieved. After the removal of duplicates, 33 studies remained for title and abstract screening. Of these, 20 studies were excluded because they were irrelevant or did not meet the eligibility criteria. The remaining 13 articles were subjected to full-text review, and 3 additional articles were excluded because of a lack of available data and comparison groups. Ultimately, 10 studies (14, 16–24) were included in this meta-analysis (**Figure 1**). Among the 10 studies, 9 studies were RCTs, one study was a case–control study, and all the studies were from China. Overall, 784 patients were included: 386 in the TIVAD group and 398 in the PICC group. The median ages in the studies ranged widely (range: 5–60 years). The outcome indices mainly included infection, catheter occlusion, phlebitis, catheter dislodgement, and thrombosis (**Table 1**).

**Table 1:** Characteristics of the included studies.

Author, year	Study design	Country	Date collection time	Group	Gender (male/female)	Age (years)	Outcome
(24)	RCT	China	2021.6-2024.5	TIVAD	22/19	49.37 ± 1.38	catheter dislodgement, thrombosis, infection,
				PICC	21/20	48.96 ± 1.29	
(14)	retrospective	China	2020.5-2021.5	TIVAD	30/18	47 ± 4.35	thrombosis, infection, phlebitis, catheter dislodgement, catheter occlusion
				PICC	33/15	78 ± 6.06	
(17)	RCT	China	2014.1-2015.5	TIVAD	23/18	45.05 ± 4.96	thrombosis, infection, phlebitis, catheter occlusion
				PICC	22/20	43.21 ± 4.79	
(22)	RCT	China	2016.7-2019.2	TIVAD	13-Dec	60.4 ± 6.0	thrombosis, infection, phlebitis, catheter occlusion
				PICC	Aug-17	58.8 ± 6.4	
(21)	RCT	China	2017.9-2018.9	TIVAD	22/24	5.4 ± 1.7	thrombosis, infection, phlebitis, catheter dislodgement, catheter occlusion
				PICC	24/22	5.6 ± 1.8	
(16)	RCT	China	2009.3-2011.3	TIVAD	17/14	35.2 ± 11.70	infection, phlebitis, catheter dislodgement
				PICC	Dec-19	34.8 ± 12.60	
(20)	RCT	China	2017.3-2018.10	TIVAD	23/20	6.35 ± 0.51	thrombosis, infection, phlebitis
				PICC	22/21	6.41 ± 0.49	
(19)	RCT	China	2015.1-2016.6	TIVAD	27/20	35.2 ± 12.50	infection, catheter dislodgement, catheter occlusion
				PICC	28/21	34.8 ± 11.70	
(24)	RCT	China	2021.3-2023.2	TIVAD	18/20	50.3 ± 7.6	infection, catheter dislodgement, catheter occlusion
				PICC	20/22	51.87 ± 7.7	
(18)	RCT	China	2013.2-2015.2	TIVAD	14-Dec	39.94 ± 6.25	phlebitis, infection, catheter dislodgement, catheter occlusion
				PICC	16/15	38.56 ± 5.51	

RCT, randomized controlled trials; TIVAD, totally implantable venous access device; PICC, peripherally inserted central catheter.

### Quality of the studies

**Figure 2:**
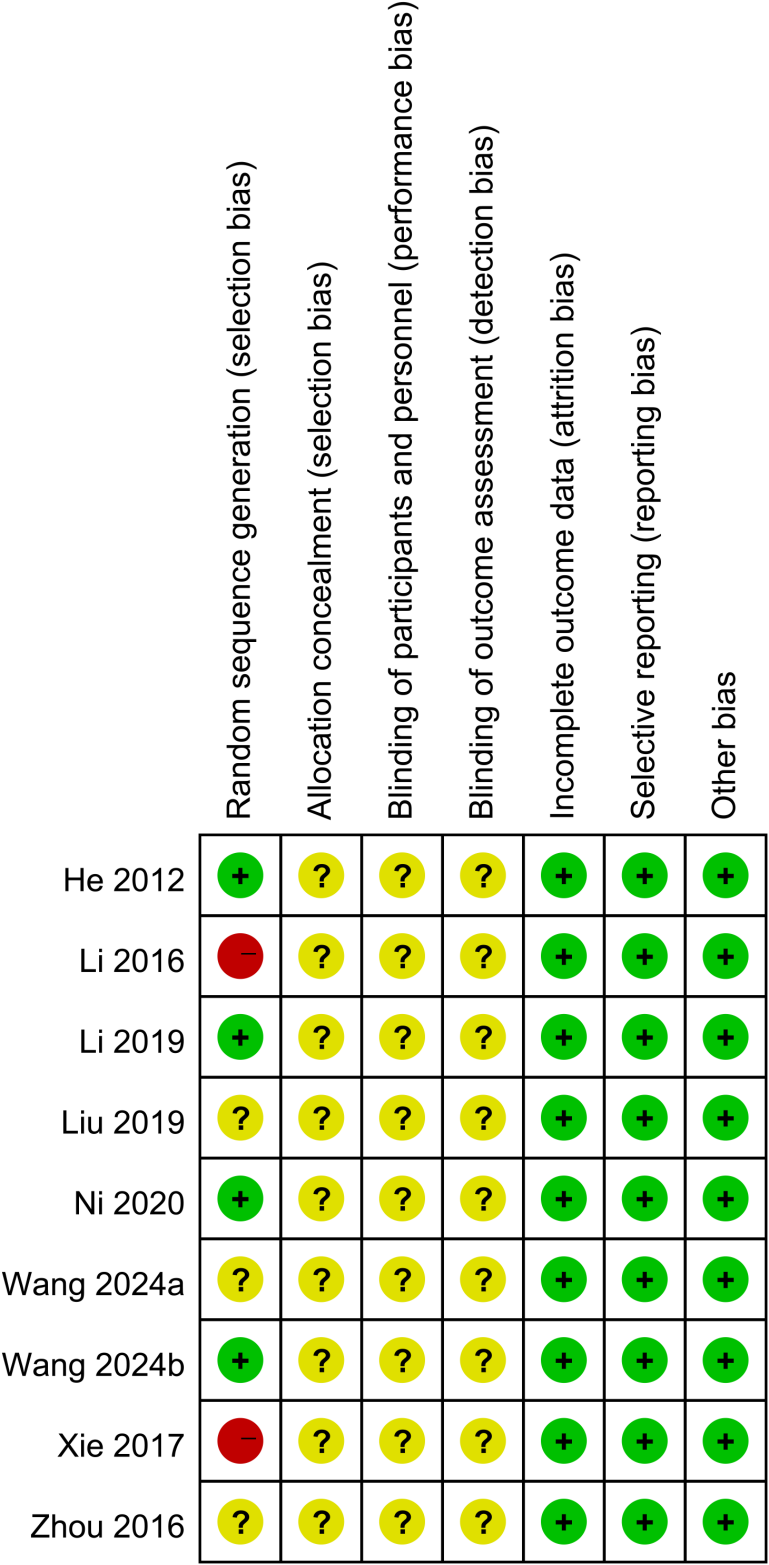
Summary of risk of bias for each included study.

Nine studies were RCTs, and the risk of bias was assessed with the Cochrane Risk of Bias tool. Two studies did not report random sequence generation. Nine studies did not describe allocation concealment, blinding of participants and personnel, or blinding of outcome assessment (**Figure 2**). One retrospective study was included, and a score of 6 was given according to the Newcastle–Ottawa Scale (NOS) score.

### Quantitative synthesis

#### Infection

**Figure 3:**
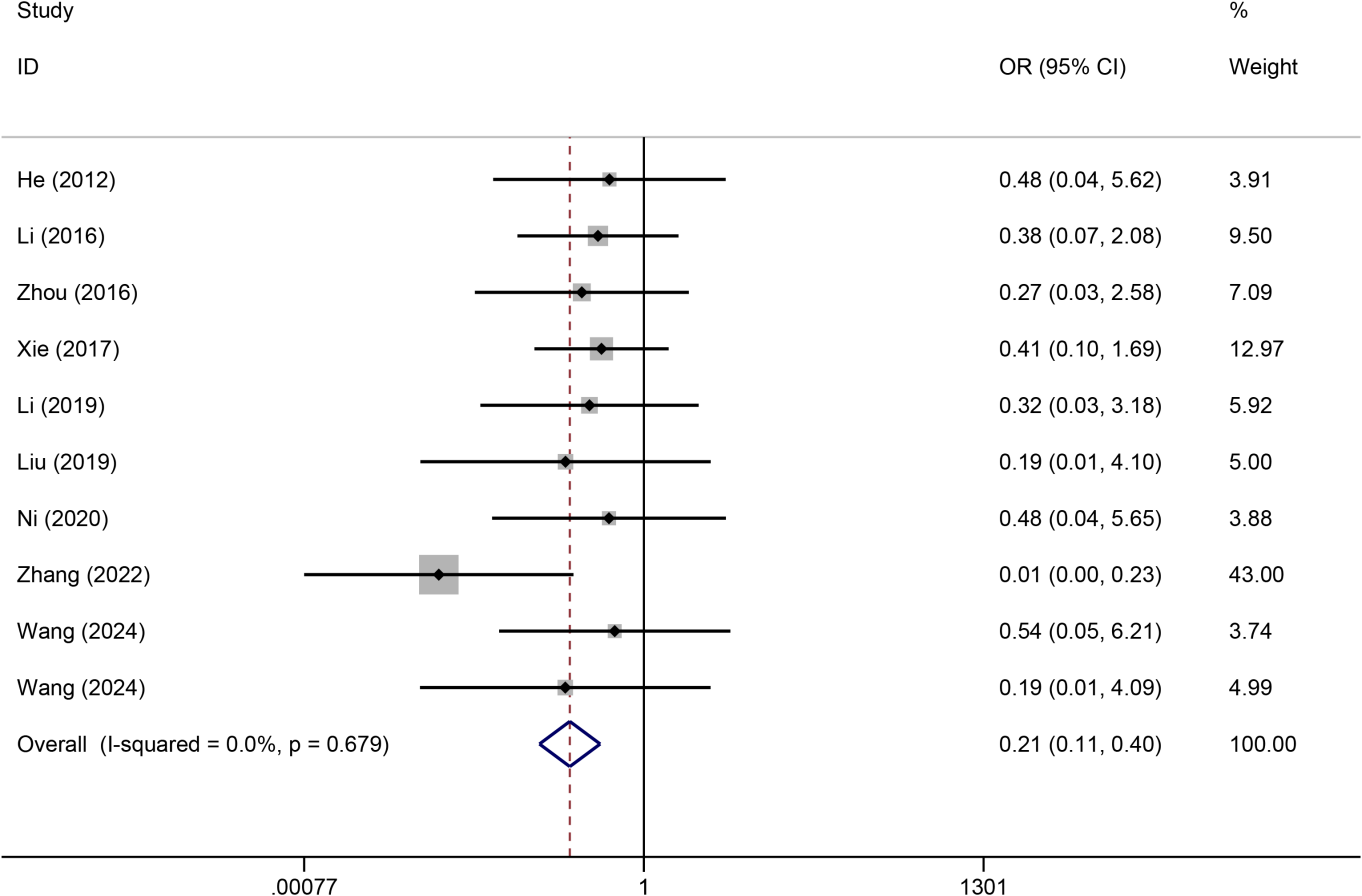
Meta-analysis of risk of infection between TIVADs and PICCs.

A heterogeneity test was conducted on the 10 included articles, and the results revealed no significant heterogeneity among the studies (I^2^ = 0%). Thus, a fixed effects model was used for the analysis. Meta-analysis revealed that compared with PICCs, TIVADs significantly decreased the infection risk (OR: 0.21, 95% CI: 0.11-0.40, p < 0.001) (**Figure 3**) (**Table 2**).

**Table 2:** The main results.

Author,year	Infection				Catheter occlusion				Phlebitis				Catheter dislodgement				Thrombosis			
	TIVAD		PICC		TIVAD		PICC		TIVAD		PICC		TIVAD		PICC		TIVAD		PICC	
	Event	No-event	Event	No-event	Event	No-event	Event	No-event	Event	No-event	Event	No-event	Event	No-event	Event	No-event	Event	No-event	Event	No-event
(17)	2	39	5	37	1	40	3	39	1	40	3	39	0	38	2	40	1	40	2	40
(22)	1	24	2	23	0	25	2	23	0	25	1	24	0	25	2	23	0	25	1	24
(16)	1	30	2	29	0	31	2	29	0	31	2	29								
(24)	1	37	2	40	0	38	1	41												
(18)	1	25	4	27	1	25	2	29	1	25	4	27	0	26	4	27				
(14)	0	48	21	27					0	48	11	37	0	48	1	47	0	48	1	47
(19)	3	44	7	42	2	45	6	43					1	46	2	47	0	43	1	42
(20)	1	42	3	40					0	43	1	42								
(21)	0	46	2	44	1	45	3	43	0	46	3	43	1	40	3	38	0	41	1	40
Pooled results (OR with 95CI%)	(OR: 0.21, 95% CI: 0.11-0.40)				(OR: 0.31, 95% CI: 0.13-0.77)				(OR: 0.16, 95% CI: 0.06-0.42,				(OR: 0.25, 95% CI: 0.08-0.76)				(OR: 0.37, 95% CI: 0.10-1.41)			

TIVAD, totally implantable venous access device; PICC, peripherally inserted central catheter; OR, odds ratios; CI, confidence intervals.

#### Catheter occlusion

**Figure 4:**
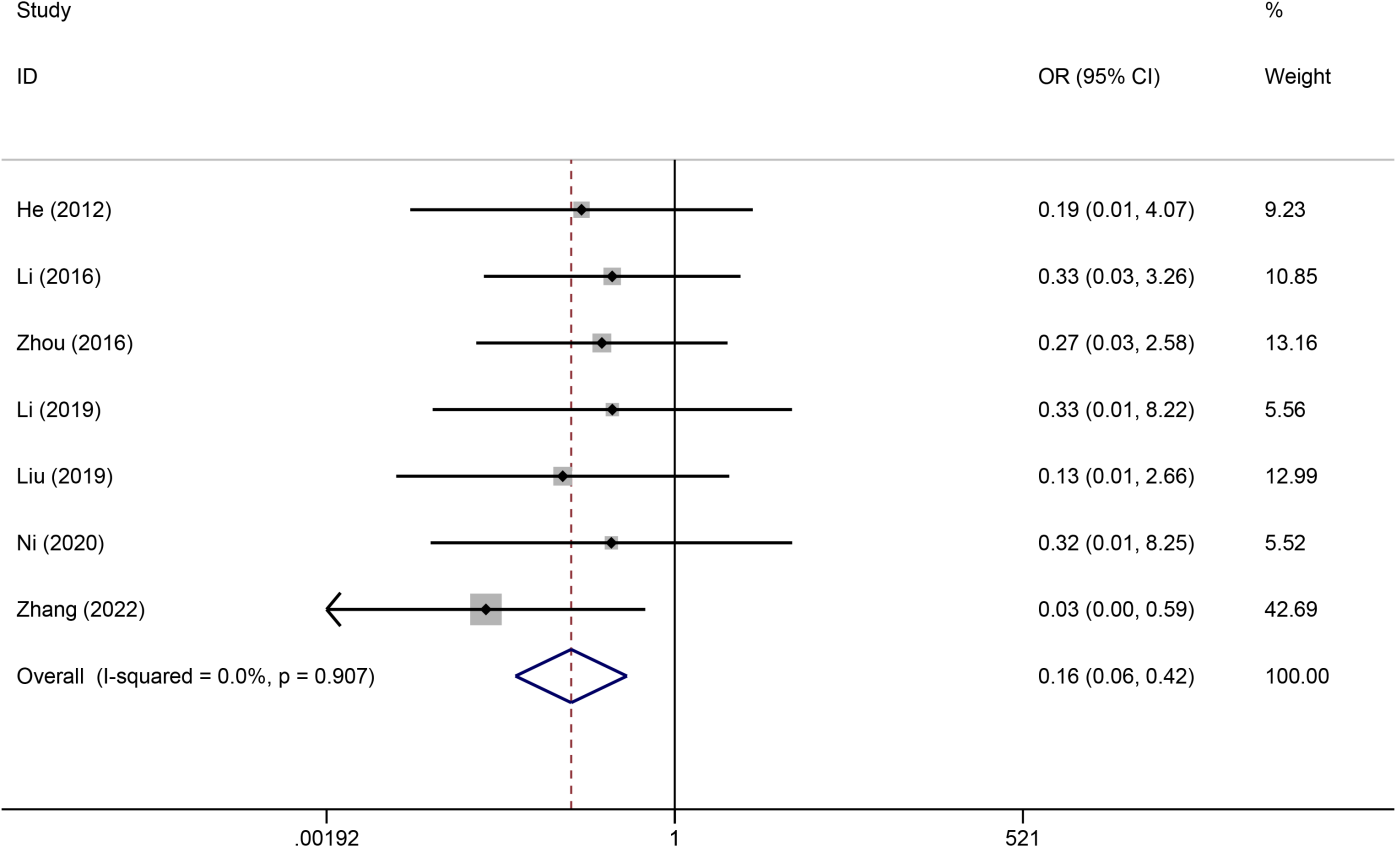
Meta-analysis of risk of phlebitis between TIVADs and PICCs.

Seven studies reported the effects of TIVADs and PICCs on catheter occlusion. No statistical heterogeneity was observed among the studies (I^2^ = 0%). Pooled analysis revealed that the risk of catheter occlusion was significantly lower for TIVADs than for PICCs (OR: 0.31, 95% CI: 0.13-0.77, p < 0.001) (**Figure 4**; **Table 2**).

#### Phlebitis

**Figure 5:**
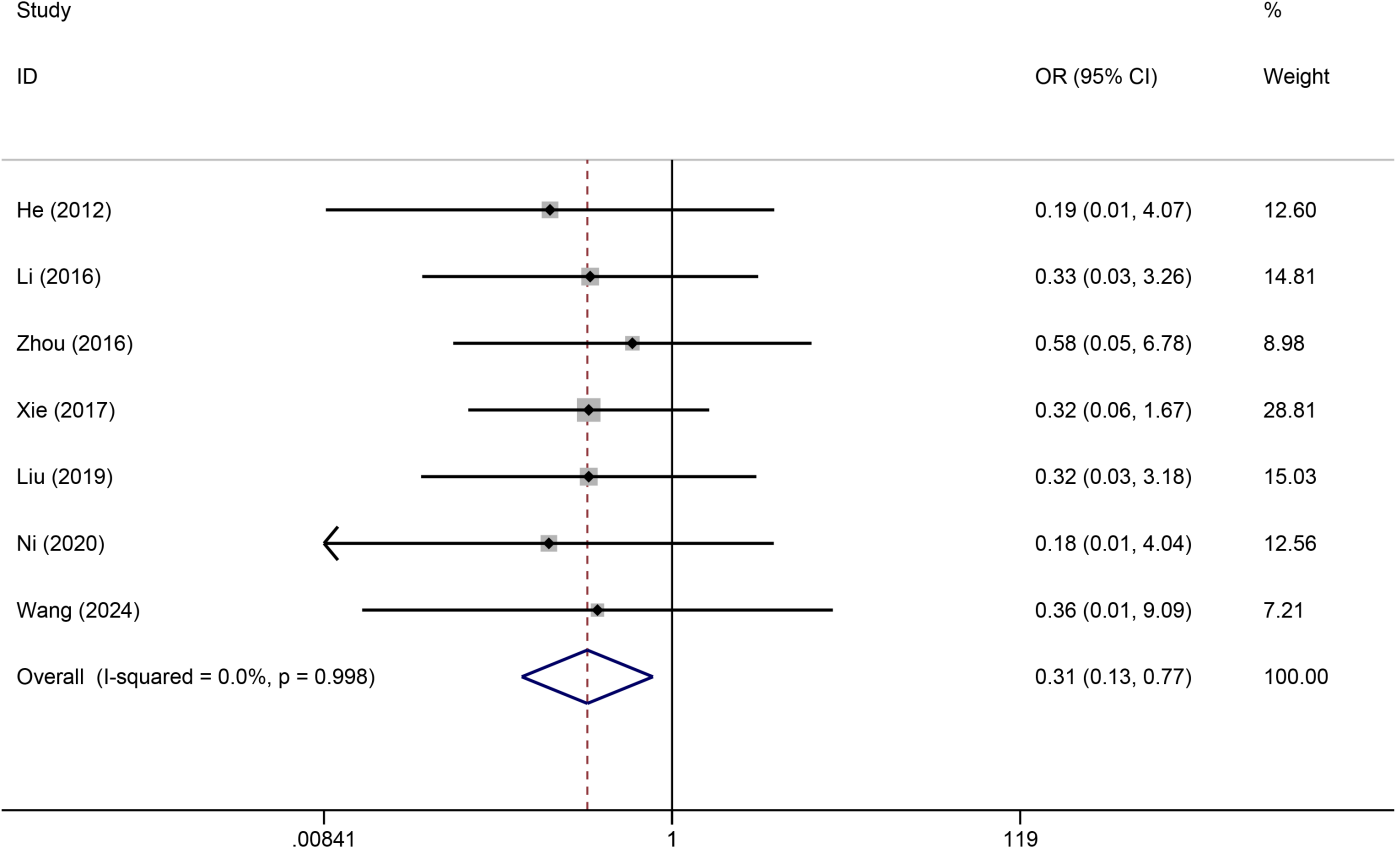
Meta-analysis of risk of catheter occlusion between TIVADs and PICCs.

Seven studies reported phlebitis data. A fixed effects model was used to pool the data because heterogeneity across the included studies was low (I^2^ = 0.0%). The results revealed that compared with PICCs, TIVADs were associated with a lower phlebitis rate (OR: 0.16, 95% CI: 0.06-0.42, p < 0.001) (**Figure 5**; **Table 2**).

#### Catheter dislodgement

**Figure 6:**
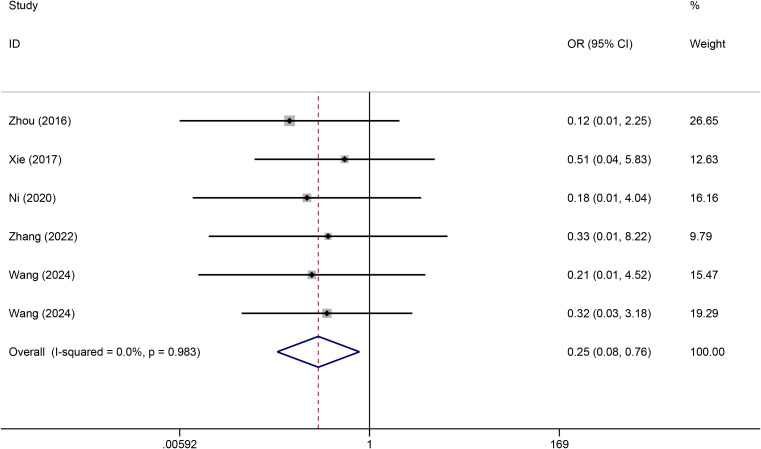
Meta-analysis of risk of catheter dislodgement between TIVADs and PICCs.

A total of six studies reported catheter dislodgement data. No statistical heterogeneity was observed among the studies (I^2^ = 0.0%). The forest plot revealed that the rate of catheter dislodgement was significantly lower for TIVADs than for PICCs (OR: 0.25, 95% CI: 0.08-0.76, p < 0.001) (**Figure 6**; **Table 2**).

#### Thrombosis

Five studies reported the effects of TIVADs and PICCs on thrombosis. No statistical heterogeneity was observed among the studies (I^2^ = 0.0%). The results revealed that thrombosis risk (OR: 0.37, 95% CI: 0.10-1.41, p = 0.144) was similar between the two groups (**Table 2**).

### Sensitivity analysis

**Figure 7:**
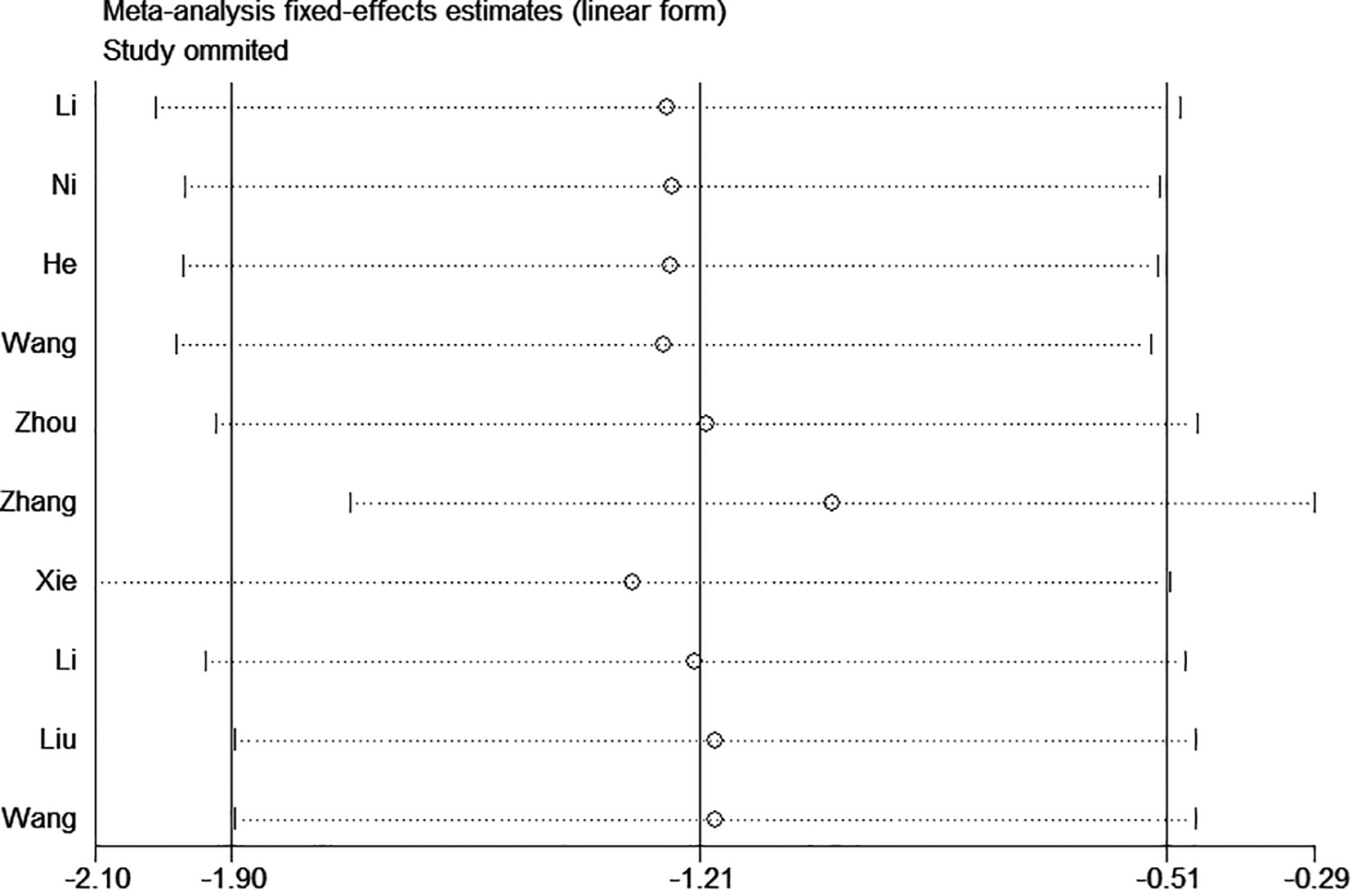
Sensitivity analysis of the impact of TIVADs on infection.

Sensitivity analyses for infection, catheter occlusion, phlebitis, catheter dislodgement, and thrombosis were performed, which demonstrated that excluding any one study did not affect the reliability of the results and suggested stability and reliance (**Figure 7**).

### Publication bias

**Figure 8:**
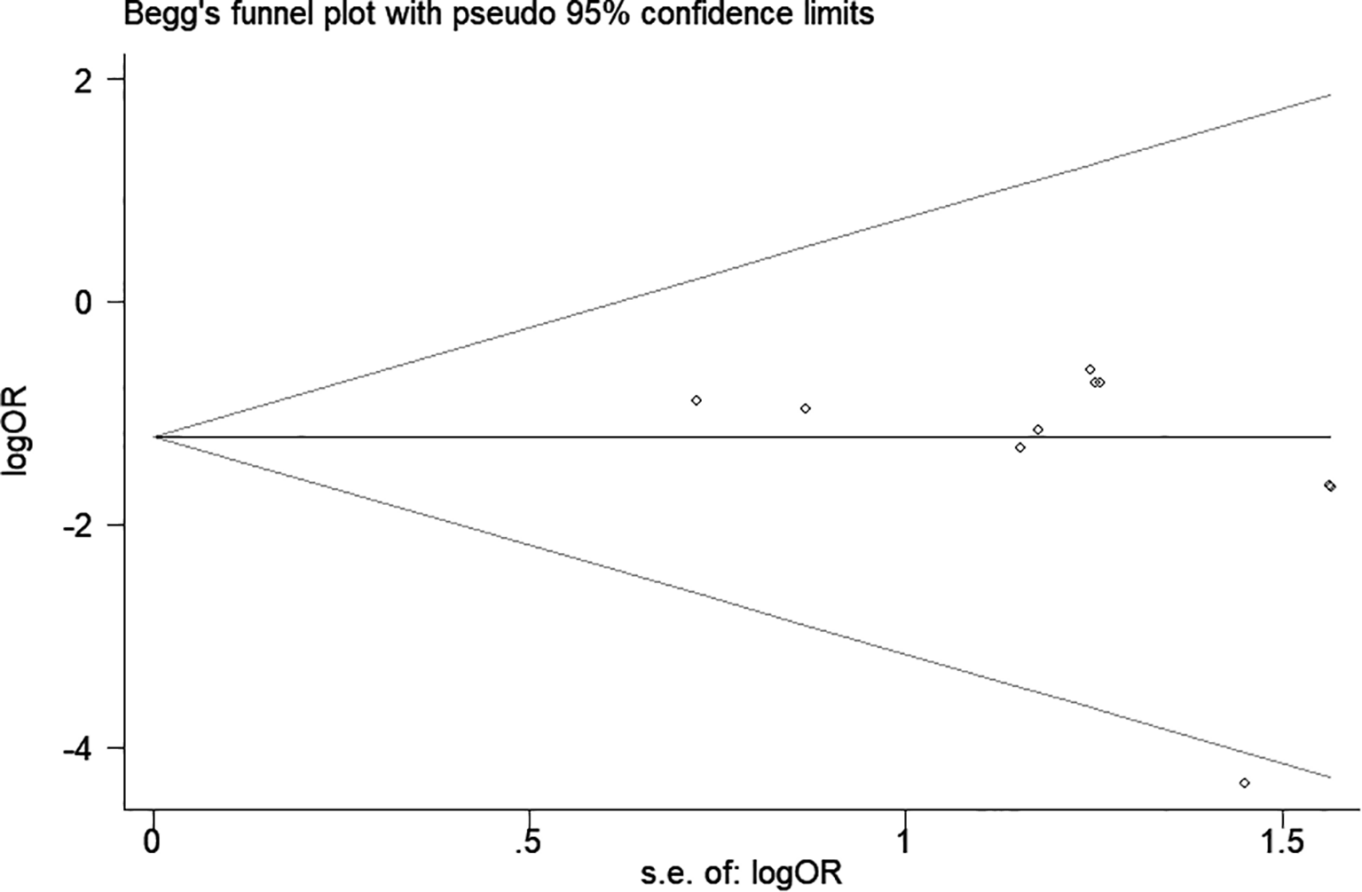
The funnel plots of the infection.

A funnel plot and Egger’s test were used to assess publication bias. As shown in **Figure 8**, the funnel plot demonstrated a relatively symmetrical distribution of studies, indicating a low likelihood of publication bias. This observation was supported by Begg’s test (p = 0.06) and Egger’s test (p = 0.192).

## Discussion

The findings of this meta-analysis provide critical insights into the comparative safety of TIVADs and PICCs in patients undergoing chemotherapy for hematologic malignancies. Our results indicated that compared with PICCs, TIVADs were associated with a lower risk of infection, catheter occlusion, phlebitis, and catheter dislodgement.

One of the most notable findings was the significantly lower infection rate in the TIVAD group. The absence of an external catheter component in TIVAD reduces the risk of contamination, which is particularly important for immunocompromised patients with hematologic malignancies (25). In contrast, PICCs have an external portion that requires frequent handling and maintenance, increasing the risk of microbial colonization and subsequent bloodstream infections (26, 27). Given that infection is a major cause of morbidity and mortality in cancer patients, the lower infection risk associated with TIVAD presents a compelling argument for its preferential use in long-term chemotherapy regimens.

Similarly, the lower rates of catheter occlusion and phlebitis associated with TIVADs may be attributed to their completely implanted nature and the materials used in their construction. TIVADs are typically made of biocompatible materials that reduce thrombotic potential (28), whereas PICCs, which remain in peripheral veins for extended periods, may trigger endothelial irritation, leading to phlebitis and subsequent occlusion (29, 30). This is particularly relevant for patients requiring prolonged chemotherapy, as frequent catheter-related complications can lead to treatment delays and additional medical interventions.

The significant reduction in catheter dislodgement rates further supports the advantages of TIVADs. Since TIVADs are surgically implanted and anchored subcutaneously, they are less likely to be dislodged because of patient movement or accidental traction (31). In contrast, PICCs, which are externally accessible, are more vulnerable to accidental removal, particularly in ambulatory patients who may be at greater risk of catheter displacement (7). This finding suggests that TIVADs may provide greater reliability and continuity in treatment, improving patient compliance and reducing the need for catheter replacement procedures.

Interestingly, our analysis did not find a significant difference in thrombosis risk between the two devices. Although previous studies have suggested that PICCs may be more thrombogenic due to their placement in peripheral veins with slower blood flow (32), the absence of a statistically significant difference in our study suggests that other factors, such as catheter materials, standardized insertion protocols, and thromboprophylaxis practices, may play crucial roles in thrombosis prevention (33). Nevertheless, the lack of detailed thromboprophylaxis data in the included studies limits definite conclusions. Future research should focus on stratifying thrombosis risk by anticoagulation protocols, catheter characteristics, and insertion methods to better inform clinical practice.

Although most studies included in this meta-analysis were RCTs, many lacked sufficient reporting of key methodological features, including randomization procedures, allocation concealment, or blinding. These omissions raise concerns about potential bias, particularly with respect to estimation of complication rates and overall effect sizes. The lack of methodological rigor may have inflated the observed benefits of TIVADs. Therefore, the results should be interpreted cautiously, and further high-quality, multicenter randomized trials are needed to corroborate these finding. However, catheter tip malpositioning is a known risk factor for PICC-related complications. Marano et al. proposed an ultrasonographic method that enables safe and accurate tip positioning without radiation exposure, offering a promising alternative to traditional radiographic confirmation (34). This technique highlights the importance of standardized, precise placement protocols in reducing procedural risks. Additionally, a recent five-year analysis by Abou-Mrad et al. demonstrated the long-term safety and efficacy of TIVADs in oncology patients, underscoring the critical role of optimized implantation techniques and maintenance protocols (35). These findings complement our results and further support the clinical value of TIVADs when used appropriately. Notably, several studies have reported immediate or early-phase benefits of PICCs (such as shorter insertion times, simpler insertion procedures, and lower upfront costs) compared to TIVADs, particularly in patients with shorter chemotherapy regimens (36, 37). However, our meta-analysis could not fully quantify these due to heterogeneous reporting and lack of sufficient data.

In addition to safety-related outcomes, patient-centered aspects such as quality of life (QoL) and ease of use are also important when comparing TIVADs and PICCs. A prospective cohort study in patients with breast or colon cancer found no significant overall difference in global QoL between the two devices, although ports were associated with more pain at insertion, whereas PICCs had a greater negative psychosocial impact (37). More recently, a large observational study in women receiving neoadjuvant chemotherapy reported that overall QoL scores significantly favored PICC-ports over PICCs, particularly among younger patients, with advantages in psychological and social domains, while device-related complication rates were similar between groups (27). Furthermore, retrospective data suggest that TIVADs may offer longer catheter dwell times and fewer removals due to complications, thereby reducing treatment interruptions and potentially improving ease of use and patient satisfaction (38). Taken together, these findings indicate that while complication rates remain essential endpoints, QoL and usability considerations should also be integrated into future comparative studies of vascular access devices.

This meta-analysis has several limitations. First, all included studies were from China, which may limit the generalizability of our results. Differences in health care systems, catheter insertion techniques, maintenance protocols, and complication monitoring practices across regions could influence clinical outcomes. Therefore, our conclusions should be interpreted with caution when applied to non-Chinese populations. Nonetheless, retrospective studies from other countries have similarly reported that TIVADs are associated with fewer complications than PICCs among cancer patients, which provides external support for our findings despite regional limitations. Second, while most included studies were RCTs, the methodological quality of the studies varied. Some studies lacked detailed reporting on randomization, allocation concealment, and blinding, introducing potential bias. Additionally, the inclusion of one retrospective study may have further influenced the reliability of our findings. Future studies with rigorous methodological designs and larger sample sizes are needed to strengthen this evidence. Third, this meta-analysis was limited to five safety-related outcomes because of the availability of data. However, other clinically meaningful factors, such as catheter longevity, patient-reported satisfaction, ease of device management, chemotherapy regimens and cost-effectiveness were rarely reported in the included studies (39). In addition, the absence of CTCAE-based grading across trials may reduce the granularity of our safety assessment and should be considered when interpreting the results. These outcomes are particularly relevant in the context of long-term cancer care, where patient comfort, treatment adherence, and health care resource utilization are critical. Future research should incorporate these dimensions to provide a more comprehensive assessment of central venous access options in oncology.

## Conclusion

In summary, our meta-analysis suggests a potential association between TIVAD use and a lower risk of complications compared with PICCs in patients with hematologic malignancies. Nevertheless, given the geographic limitations of the included studies, the absence of cost-effectiveness assessments, and the moderate overall sample size, these results should be interpreted with caution. Additional large-scale, multicenter studies in diverse health care settings are required to validate these findings.

## Data Availability

The original contributions presented in the study are included in the article/supplementary material. Further inquiries can be directed to the corresponding author.
